# Shared Situational Awareness in a Professional Soccer Team: An Explorative Analysis of Post-Performance Interviews

**DOI:** 10.3390/ijerph17249203

**Published:** 2020-12-09

**Authors:** Gaute S. Schei, Rune Giske

**Affiliations:** 1 Department of Sports Science and Physical Education, University of Agder, 4630 Kristiansand, Norway; 2 Department of Education and Sports Science, University of Stavanger, 4021 Stavanger, Norway; rune.giske@uis.no

**Keywords:** team performance, coordination, situational assessment, qualitative interview

## Abstract

Sport science research has done little to elaborate on the cognitive factors that turn a collection of individual players into a coordinated elite team. The purpose of this paper is to clarify if the players and coach of an elite soccer team express shared situational awareness. Ten players and one coach were exposed to twelve video pictures from a previous soccer match, and their statements for each picture were recorded and analyzed using a qualitative approach. Two of five game situations were with ball possession and three out of seven were without ball possession; the player statements are contradictory, with a high threat for inadequate coordination. In seven of the twelve game situations, the players’ statements coincided and expressed a shared situational awareness, with good opportunities for adequate defensive and offensive coordination. In two of the game situations, there was a high threat for inadequate coordination. There was consensus among 9 out of 10 players, but the player with the divergent statement was central in the situation. The procedure followed in the study could be used to elucidate if a team has shared situational awareness and clarify in which situations there exists discrepancies and data that can be used to improve team coordination on and off the field.

## 1. Introduction

Soccer teams can be described as action teams, where performance is characterized by rapid, complex, and coordinated task behavior [1], and where the team dynamically adapts proactively and reactively to the environments within which they operate [2]. The players’ actions create a continuous stream of playing situations, where the effect of an action depends on the actions of other actors. Coordination between team members becomes therefore critical, considering how the team as an entity dynamically solves defensive and offensive tasks, where different team members primarily undertake different tasks [3]. Hence, team cognitive properties were considered by several authors to be a team performance prerequisite [4,5]. Therefore, elite soccer players must acquire team competencies that include requisite knowledge, principles, and concepts underlying the team’s performance [6]. Collective guidelines of the players’ perception, what Collins and Collins [7] refer to as the master plan of the coach, are task-solving cursors that can be conceptualized as a shared mental model (SMM). Cannon-Bowers, Salas, and Converse [8] describe the SMM as knowledge structures held by members of a team that enable them to form accurate explanations and expectations of the task by coordinating their actions and adapting their behavior to the demands of the task and other team members. In basketball, Phil Jackson, the legendary coach of the Chicago Bulls and Los Angeles Lakers, has an offensive system named “the triangle”, which can be considered as a shared mental model of task sharing. Jackson describes the “triangle” as “five-man tai chi”, because it involves the players moving together in response to the way the defense players position themselves [9]. In other words, the shared knowledge makes it possible for the players to move together in unison so they can take advantage of openings the other team’s defense offers. The purpose of the SMM is to permit team members to draw their own structured knowledge as a source for choosing actions that are consistent and coordinated with those of their teammates, and as the level of task interdependence increases, teams rely more on team member coordination as a central process for effective functioning [10].

Shared cognitions have primarily been studied outside of sports, and a review of the literature has shown that there is a strong, positive relationship between team cognition and behavioral process, motivational state, and performance in military, educational, medicine, industrial, and high-tech settings [11]. In the last two decades, there has been emerging interest in sport science, and the SMM has been explored in collegiate basketball teams [12], soccer teams [13,14], rugby officials [15], ice hockey, and handball teams [16,17]. Research shows further that a lack of shared knowledge could lead to weak coordination and reduce the team’s ability to adapt to changing environmental demands [8], and teams experiencing communication breakdown are more likely to experience difficulties with coordination [18]. In an elite team sport setting, Apitzsch [19] showed that two out of five major factors that lead to a collective collapse in handball were failure of the role system and negative communication within the team. Reimer, Park, and Hinsz [20] propose that assigning players to particular roles should enhance coordination and team performance, because the ambiguity concerning who is doing what is reduced. Giske and colleagues [17] underpinned this assumption when they demonstrated a strong positive relationship between role clarity and the SMM in elite team handball and ice hockey. Moreover, an understanding of what to do and when to do it are a precursor to a mental model of task sharing.

To date, SMM research on interactional team sports has shown that shared knowledge among players could probably promote better understanding of players’ synchronized actions during the game. Salas, Stout, and Cannon-Bowers [21] suggest that there is a positive relationship between shared knowledge and team situational awareness, and propose that information that is shared in strategic models allows members to have common explanations of the meaning of the task cues, as well as make compatible assessments of the situation and form common expectations. Endsley [22] simply states that individual situational awareness is knowing what is going on around the individual, whereas Wellens [23] expands the definition to group situational awareness, defining it as “the sharing of a common perspective between two or more individuals regarding current environmental events, their meaning and projected future status” (p. 272). In other words, what to do and when to do it in unison in interactional team sports presuppose shared situational awareness. In sports, however, Eccles and Tenenbaum [5] develop a “flower” model illustrating knowledge unique to each team member and general and specific knowledge shared by multiple team members. Bourbousson, R’Kiouak, and Eccles [24] adapt and refine this model to cover situational awareness, and suggest that team members’ connections are numerous and local, indicating that dyadic or triadic arrangements of players are well-connected, where one player heeds the other’s actions. In a team setting, common interpretation of cues or overlap of each member’s individual level of situational awareness allow for action that is both accurate and expected by teammates [21,22,25]. On the other hand, insufficient shared awareness could lead to weak coordination, and reduce the team’s ability to adapt to changing environmental demands. It was further suggested that repeated experience in an environment allows one to develop expectations about future events, and introduces immediate pattern-matching mechanisms as fundamental for developing situational awareness [22]. Klein [26] claims that once a situation is assessed, the appropriate course of action will usually be apparent without deliberation, indicating that assessing a situation and retrieving information on how to deal with it are part of the same process, something known as recognition-primed decision-making. In line with this idea, Eccles and Tran [27] suggest that practice sessions and games in sports provide opportunities for team members to acquire situational probability related to their own team and individual teammates.

To our knowledge, few studies have been empirically concerned about the players’ shared situational awareness, which in SMM theory is considered essential to establish synchronized team behavior. Bourbousson, Poizat, Saury, and Sève [28] interviewed five basketball players when they viewed a videotape of a previous 10 min real match. The results from the post-match interviews show that the same typical concerns were relatively rare, but partial sharedness occurred more frequently and the same typical concern was evoked when they all recognized the same situation type. Bourbousson, Kiouak, and Eccles [24] revealed in a similar basketball research design using a social network analysis that team members had a low level of awareness of their teammates, and that one team member in each team often heeded or was heeded by his teammate, indicating that some players appear more important in coordinating the team. Therefore, several authors argue that descriptive and empirical studies are needed to improve our understanding of how team sports function [4,29,30]. However, most of the research on SMMs has been non-empirical [5,20,31], or done by questionnaires [16,17] and interviews [13]. Previous post-performance interviews with video exposure are primarily conducted in youth basketball [24,28]. Therefore, the purpose of the present study is to gain insight into the shared situational awareness of a professional soccer team and their coach by exposing them to a video from a real match where all participant players were in the line-up.

## 2. Materials and Methods

### 2.1. Research Design

Yin [32] argues that the case study is an especially appropriate research strategy if the researcher wants to understand a phenomenon in depth and within its real context. Investigating a professional soccer team can be considered an extreme case, which is a research strategy that generally follows an idiographic approach [33], and often reveals more information and more basic mechanisms in situation studies [34]. Classical case studies usually focus on an individual person as the case, but the research methodology is also applicable to small groups [32]. The research design is exploratory, and two types of data were gathered: (a) video recordings of the team performance of a real match, and (b) verbalizations during post-performance interviews. Cook and colleagues [35] recommend process tracing techniques, which are methods for collecting data concurrently with task performance, as an approach to gather data about knowledge underlying task performance. Thinking aloud during performance was considered as a methodology for gathering process data, but it strongly interferes with the players’ task completion. Direct access to process data that reveal something about knowledge heterogeneity in an elite soccer team is therefore challenging, indicating that the research design in the present study is probably the most appropriate knowledge elicitation method.

Based on primary aircraft studies, Endsley [22] developed the situational awareness concept, suggesting different zones extending outward in time and space from the individual (i.e., immediate, intermediate, and long-term), where the immediate surroundings seem to be the most relevant for synchronized group behavior in soccer. Yin´s [32] argument is that a real-world case study assumes that important contextual conditions are pertinent in the case. Since context-dependent knowledge appears as the heart of expert activity [34], professional soccer players’ verbalization of given game situations could reveal the degree of shared awareness in the team. Therefore, participants in this study were exposed to twelve video situations from a previous match and were questioned about those situations. Based on the individual player’s and the coach’s statements, the analysis intended to explore shared cognition in selected game situations, and this procedure was inspired by the suggestions of Eccles and Tenenbaum [5] related to the measurement of shared knowledge in sports, and Bourbousson, Kiouak, and Eccles’ [24] study of a basketball team. Furthermore, Cooke and colleagues [35] argue that a holistic approach to team knowledge measurement requires new methods; for example, interviewing the team as a whole. A basic assumption in this holistic approach is that team knowledge is more than a collection of aggregated individual team member knowledge. Cooke with colleagues [35] argue that we need knowledge elicitation methodologies that address a more fleeting, context-specific understanding of a situation.

### 2.2. Participants

An elite professional soccer team consisting of 10 players and their head coach volunteered to participate in the present study. The participants were 26.1 (SD = 4.7) years old, with playing experience on the same team of 4.27 (SD = 4.3) years. Eight players were in the line-up most of the games in the season. A criterion for inclusion was that the players were in the starting line-up in the match where the exposed video sequences were recorded. The study was conducted according to the Helsinki Declaration and the Norwegian National Committees for Research Ethics. This study is approved by the Norwegian Center for Research Data (id: 738807). Table 1 shows the participants´ characteristics in detail.

### 2.3. Video

Soccer is an intermitted game where the ball is in and out of play, and research has shown that effective playing time in the World Cup final is decreasing. In 2010, it was approximately 52% of match time [36]. The game dynamically changes, and situations grow and disappear continuously, primarily because of the distribution of ball possession between the opponents. Twelve videos out of this stream of situations were selected from a match, seven situations while the team was not in possession of the ball and five situations while in possession of the ball. Bergo, Johansen, Larsen, and Morisbak’s [37] categorization of playing situations formed the basis of the selected situations. Therefore, there were four situations in established defense (when the team has balance, is in control of threatening spaces, and has enough players on the right side of the ball); three situations in defensive transition (the team has lost the ball, there is an imbalance between the ball and the team’s own goal, and the players are not in control of threatening spaces); four situations in established attack (the attacks start with ball possession in the rear or central parts against a team with good defense balance); and one situation in offensive transition (the team captures the ball and the opponent is in defensive imbalance). All situations were taken from the first half of the game, and the score was 1−1. One of the three defensive transition situations resulted in a goal against the examined team.

### 2.4. Procedure

A video camera (Canon XA20, Tokyo, Japan) was used to record participants’ statements and, in addition, a tape recorder was used to ensure voice documentation. The playing situations were exposed by a projector (NEC np-m300w projector, Tokyo, Japan) on a canvas. All technical equipment and appropriate facilities were made available for the research team from the club at the club stadium. After each exposed video picture, the player was simply asked: “Describe what you perceive in this situation?” Depending on the player’s answer, different follow-up questions were utilized to add nuance to, concretize, and explore all (newly) mentioned sources of information in the players’ statements [38]. In addition to these questions, probes were made to create a natural and effortless conversation with the subjects [39]. After video sequences, the players were asked if they perceived that the team in general was guided by a shared understanding during matches, and if so, in what way. The interview length among the players varied between 27–42 min or 7–14 transcribed pages (Table 1). A basic assumption in this study was that elite soccer players are experts that are able to verbalize their continuing thoughts [40] when exposed to the video of their team in a game situation where they might be centrally or peripherally localized. A similar research design was used in basketball [4], soccer, and team handball [41]. However, there is most likely unconscious information from the game situation encounters with teammates that the player is unable to verbalize that might be crucial in co-acting. These elements are left unaddressed in the analysis.

### 2.5. Data Analysis

Endsley [22] and Bourbousson and colleagues [24] suggest that team situational awareness can be visually illustrated by overlapping circles between individual team members’ situational awareness, and they propose that this knowledge state may serve as an index of team coordination. According to Eccles and Tran [27], team coordination is the process of arranging team members’ actions (type of action, timing, and location) so that, when combined, they are in a suitable relation for the most effective result. The data corpus was therefore analyzed to reveal similarity or differences between players’ perception of each of the twelve videos. Contradictory statements between players are considered a threat for inadequate coordination, while similarity provides good possibilities for adequate coordination. The criteria used were the situational description (theme—terminology—playing area—positions) and situational solution (e.g., “We have complete control in this situation. They have one player in the box and one that is on his way to the box. We have 5–6 players located in the situation, so we are playing six against three. We should have complete control, but player 4 has the totally wrong position.”). This situational description and solution stating that player 4 has the wrong position and that the team is in the numeric majority were held by eight of the respondents. An example of contradictory statements is the following: “we shall defend in zones” and “we shall defend man–man”. The number of players with contradictory statements was considered a greater threat for coordination. The location (central or peripheral) of the player in the situation and coordinative solution were also emphasized in the analysis (e.g., in situation 9, the player with the ball had a deviant statement compared with the others, and because he is in possession of the ball (central), it became a threat for inadequate coordination). All transcripts were conducted using NVivo software (version 11.1.0.411) (QSR International, Burlington, NJ, USA) for organizing the qualitative data. The analysis was organized based on defensive pictures (the opponent has the ball in possession) and offensive pictures (investigated team in possession of the ball), and to avoid subjectivity, all authors were involved in the analysis.

## 3. Results

Table 1 shows that the length of interviews diverged considerably, and this may reflect different abilities to articulate answers or verbalized meaningful information when the players are exposed to the videos. Years of experience, number of matches, and experience from national matches seem to be unrelated to the quantity and quality of the interviews.

When the respondents were asked if they experienced that the team was influenced by a shared mental model, some of the players were unsure about the question. However, most of the players and coach argued that the defensive part (pressure on the player with the ball and right positioning) was more pertinent and somehow easier, and it was more challenging to create a shared understanding in the offensive part of the teams’ behavior. Interestingly, one of the players specified changes in position, playing formation, and turnovers as obstacles to building shared cognition in the team.

**Figure 1:**
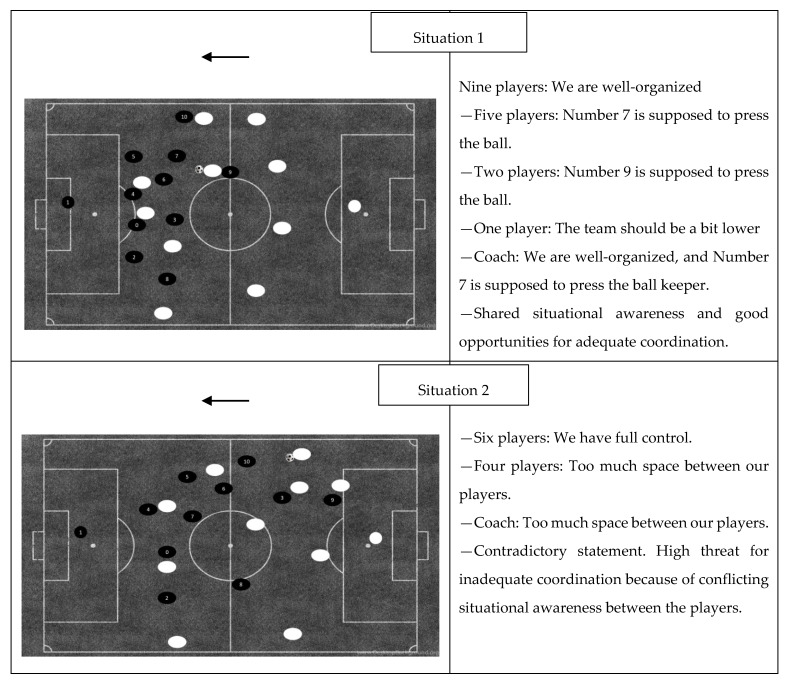

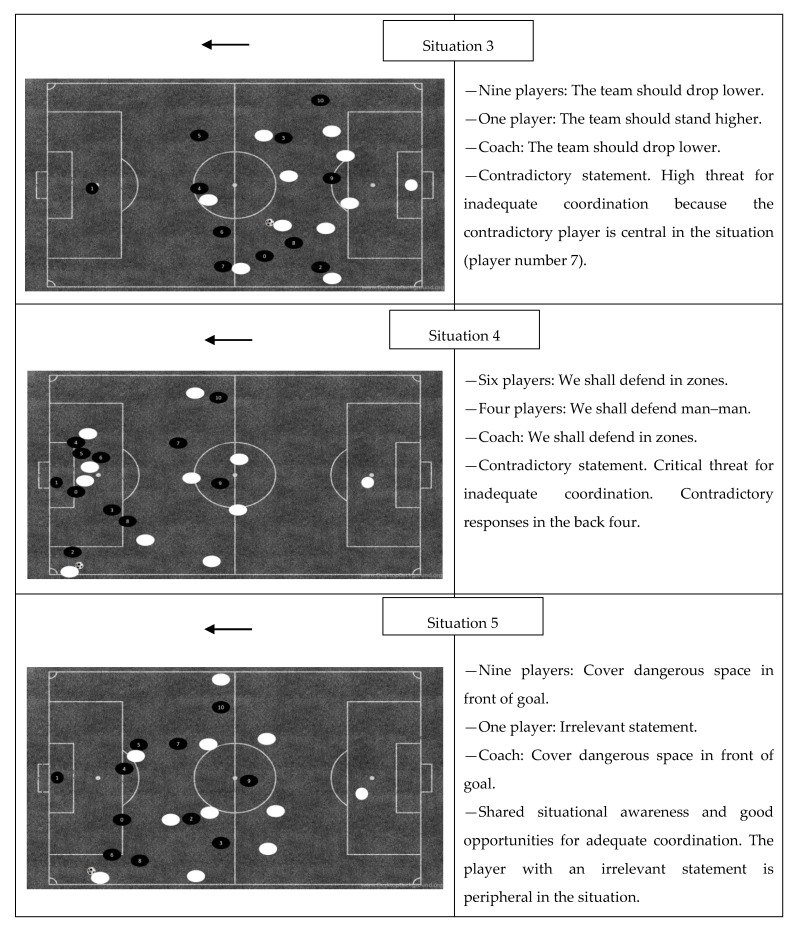

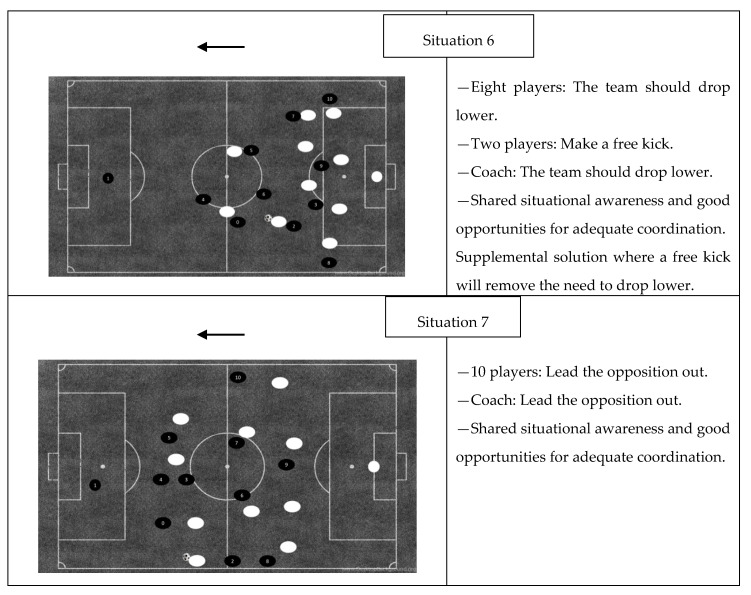
Defensive situations and the distribution of player responses in categories. Players with numbers represent the inquired team and the arrow specifies the attacking direction. Player 0 is the player that did not participate in the study.

The results displayed in Figure 1 show the compliance of the players’ (and coach’s) statements in seven game situations where the opponent had ball possession. In three of the situations, the players’ statements were contradictory, with a high threat of inadequate coordination. There was a consensus among 9 out of 10 players in situation number three, but there was still a high threat of inadequate coordination because the player with the divergent statement was central in the situation. In four of the exposed defensive situations, the statements expressed shared situational awareness and good opportunities for adequate coordination.

**Figure 2:**
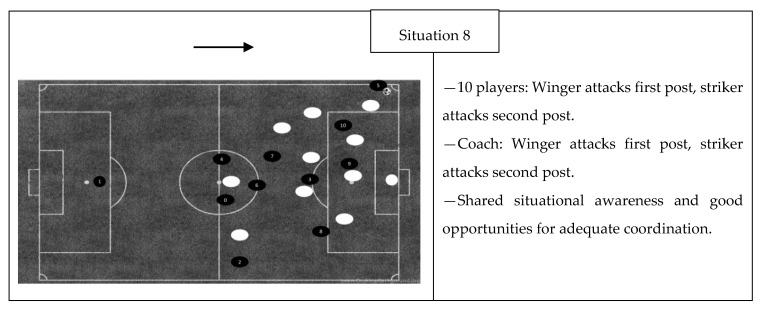

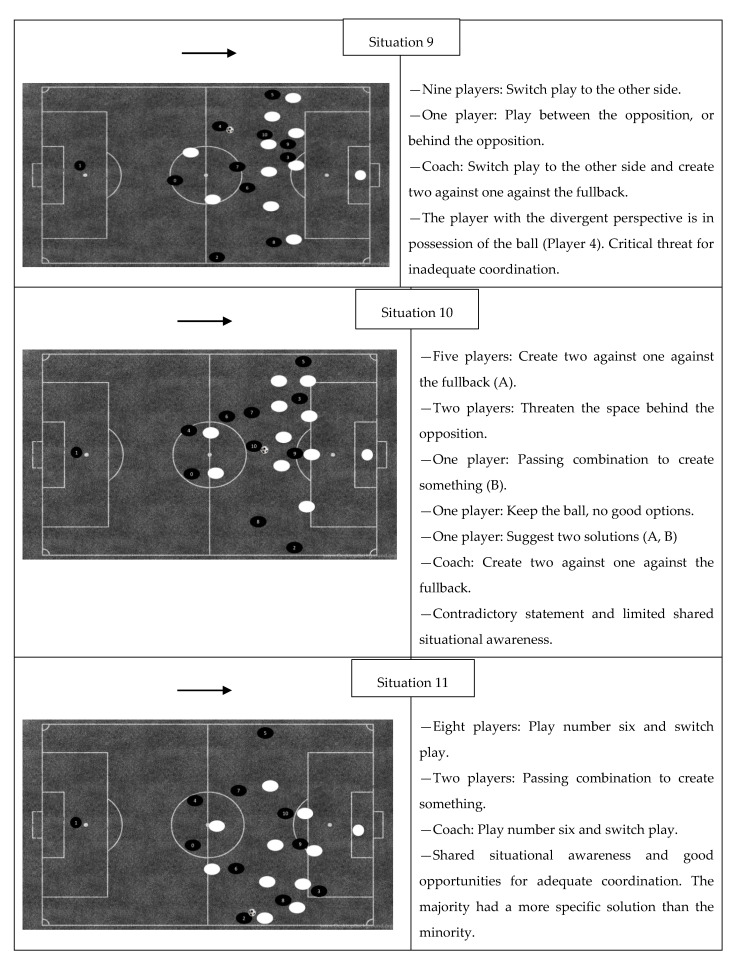

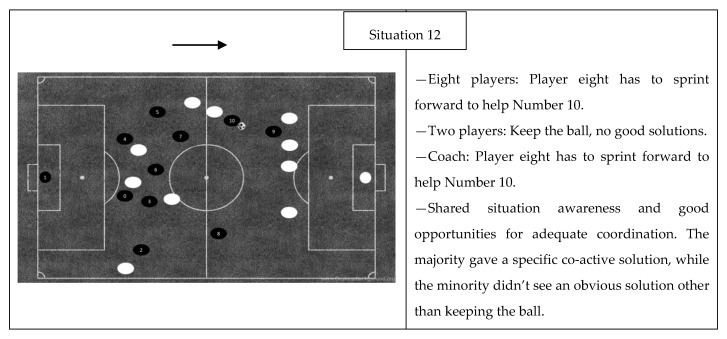
Offensive situations and the distribution of player responses in categories. Players with numbers represent the inquired team and the arrow specifies the attacking direction. Player 0 is the player that did not participate in the study.

The results displayed in Figure 2 show the compliance of the players’ (and coach’s) statements in five game situations where the examined team had ball possession. In three of the situations, the players’ statements coincided and expressed a shared situational awareness and good opportunities for adequate coordination. In two of the situations, where the team was in possession of the ball, the player statements were contradictory with a high threat of inadequate coordination. There was a consensus among 9 out of 10 players in situation number nine, which should indicate sufficiently shared situational awareness, but there was still a high threat of inadequate coordination because the player with the divergent statement was central in the situation.

## 4. Discussion

The purpose of presenting an empirical study is to gain insight into the shared situational awareness of an elite soccer team and their correspondent coach by exposing them to videos from a previous match. Endsley [22] argues that there is evidence that a person’s manner of characterizing a situation will determine the decision process of solving a problem, and that every team member must have situational awareness for all of his or her requirements or will risk becoming the proverbial chain’s weakest link, independent of overlap demands. Eccles and Tenenbaum [5] claim that similar knowledge is required to establish team coordination, and an increased number of equivalent player statements related to the individual exposed video should therefore be desirable. The results from Figure 1 and Figure 2 reveal variegated depictions, and show that the verbal statements from the players in seven of the exposed game situations correspond in such a way that the team has sufficient shared situational awareness and therefore good opportunities for adequate coordination. Interestingly, Bourbousson with colleagues [4] argue that the coordinated network was quite hetrogenus, and essentially built on local coordination where one player heeds the co-action in such a way that the team does not necessarily form a single unit. However, to have knowledge and to understand that the players’ task in this situation is not to move or make an initiative is also a vital part in the process of appearing as a unit. Local coordination on this performance level presupposes that the players that are not directly involved do not interfere.

In five of the exposed game situations, the comparison of the verbal statements reveals contradictory situational awareness, and there is a critical threat for inadequate coordination. The players’ responses in situations two, four, and 10 reveal a minority group of four players or five players, and in situations three and nine, one centrally located player expresses a contradictory point of view compared with the rest of the group. Based on Eccles and Tenenbaum’s [5] assumption that similar knowledge is required when establishing team coordination, an increased number of deviant statements are undesirable, and it becomes more critical if the players are central in the coordination solution. Salas et al. [25] argues that a complete overlap is probably not the most expedient, because it is time-consuming to establish and preservative in the sense that it reduces the availability of solutions. They suggest that each member is required to have sufficient similar and compatible mental models guiding them towards team objectives. These five situations, which are assessed with a high threat for inadequate coordination, have contradictory verbal statements and incompatible solutions from relevant players in the game situation. In the remaining seven exposed videos, the players’ statements and their localization reveal sufficient overlap, and their shared awareness of the situation might influence their individual decision process in such a way that it enables efficient team coordination.

The analysis also has considered the players’ locations in the game situation when the threat for inadequate coordination is assessed. Since previous findings have primarily been obtained in basketball, such as Bourbousson with colleagues [4,24,28], where the playing area is smaller and the number of players fewer, the localization of the player in game situations appears more significant in coordination in soccer. In situation three, the player that held a contradictory point of view compared with the rest of the team members could lead to coordinative difficulties because he has a central position in that situation. This is not the case in situation five, where the statement from the deviant player is characterized as irrelevant because he is localized as peripheral in the situation. Even though there is only one player in both of these situations who has a statement that deviates from the rest of the team, this deviation could have a negative effect on team coordination [20]. Contradictory statements among players that are central in the situation are considered by far more devastating for team coordination than a blurred statement from a peripherally located player.

The findings underpin the dynamics of shared situation awareness among team members, and designate great demands on continuously monitoring and updating the situation to enable the players’ coaction. Previous observational research on elite soccer players shows that higher frequencies of head movements (explorative actions) are positively related to the individual player’s game performance [42,43,44,45]. The shared situational awareness perspective in the present study complements this individually player-oriented research avenue, and points out that perception in elite soccer also has an essentially collective dimension and how the team as an entity dynamically solves defensive and offensive tasks [3]. However, these overarching de-contextual defensive and offensive tasks must be further elaborated on by establishing mutual expectations in different offensive and defensive game situations before they can promote internal predictability, which is critical in co-acting [8]. Because the behavioral expectations are so closely related to game situations (the players’ task is more or less continuously defined by the situation), providing equal or approximately identical situational assessments becomes an important prerequisite for coordinated player behavior in an elite soccer team.

Previous research on team coordination has not been particularly concerned with the distinction between defensive and offensive situations, despite the fact that the defensive part of the game (the team is not in the possession of the ball) seems to be more reactive, while the offensive part can be considered more proactive. Comparing the results displayed in Figure 1 and Figure 2, both offensive and defensive situations reveal comparable statements with good opportunities for adequate coordination and contradictory statements that pose a high threat of inadequate coordination. This finding indicates that shared situational awareness in an elite soccer team is a coaching issue for both offensive and defensive situations. However, the answers from the open questions and responses from the exposed videos reveal that there are differences between the players’ responses comparing offensive and defensive situations. The defensive situation evoked more contradictory statements, while differences in offensive situations seem to be more aimed at the degree of specificity (e.g., situations 11 and 12). Established attack and offensive transition situations may reduce available solutions with a detailed coordination plan that impairs team effectiveness, because the idea is often to take the opponent by surprise, which means a higher risk [37]. Defending against established attack and offensive transitions may be considered as more reactive with fewer solutions, lower risk, and defined backup behavior. Shared coordinated solutions in defensive situations thus become more expedient.

Cannon-Bowers and Salas [46] categorized the content of shared mental models in task and team member knowledge, and previous qualitative research among elite soccer players reveals that knowledge about teammates’ strengths, weaknesses, and preferences in specific situations are important as a source in the decision-making process during the game [20,47]. The exposure of one of the offensive videos (12) awoke a response among seven of the respondents related to team member knowledge. Player three expressed: “We know that player 10 is extreme in the one against one situations”, and player five said: “Then he will be set up in situations where we know that he is good”. There are several aspects of the responses worth commenting on. First, player three uses the plural pronoun “we”, which indicates that this knowledge is shared in the team. Secondly, player five’s responses show that the team endeavors to create situations where Number 10 can display his special skills. This finding seems to be in line with Giske et al. [47], suggesting that in games like soccer, it is possible to create a situational development that gives team members an opportunity for pattern recognition. Furthermore, it shows how interviewed team member knowledge is in specific game situations. Previous research has primarily been concerned with situational awareness, more like monitoring or visual search [42,48], but, in elite team sports, it is also about creating situational conditions that provide a chance for pattern recognition, preferably without disclosing the intention to the opponent [47]. This is about the difference between the ability to see opportunities and the ability to create opportunities in the game.

Video number four is a cross-defensive situation, and the responses reveal the most conflicting viewpoints among the players in the squad. Most of the players and the coach say that the team shall defend cross situations by zone organization. However, four of the players express that the team shall defend these situations by man-to-man marking. Two of these four players are defenders, where one of their primary tasks is to solve these situations. One of the players (number four) stated: “We haven’t discussed so carefully if we are organized in zone or man-to-man marking. I think zone, but obviously you attack the ball and mark the man in your zone.” The quote is complex, and can be interpreted in several different ways, but in this context, the most obvious is that he is unsure of the solution because it has not been accentuated in the coaching process. In other words, he makes a reservation before he states his point of view. This reservation supports the findings by showing conflicting differences between the players’ points of view, and indicates that there is no accurate shared knowledge in the team concerning how this situation should be solved [8]. This finding reveals a potential for a coordination breakdown [27], and should therefore be accommodated by a teaching sequence.

Mutual performance monitoring has been defined as the ability to keep track of fellow members’ work to ensure that everything is running as expected and, in addition, ensure that they are following procedures correctly [49]. According to Eccles and Tran [27], effective mutual performance monitoring requires shared knowledge of the task responsibility, and they suggest that if the team does not share the same mental model for how the team should appear, performance monitoring becomes ineffective. The findings when the players were exposed to video number five show a high degree of agreement about the major task in the situation (cover dangerous space in front of goal), but a closer inspection of the data also reveals that six of the respondents claim that the team has a feeble marking in a cross situation, and that there is a shared understanding among them that the team has not perceived the situation well enough. These six players also share a common monitoring of team coordination in the situation, which presupposes a shared knowledge of an ideal coordinative solution. The players’ responses to video eight show that all of the respondents (the coach included) monitored the situation in the same manner, and they suggest the same team solution to solve the situation. According to Salas et al. [25], the SMM is important for mutual performance monitoring, as it provides co-players with an understanding of what team members are supposed to be doing in a given situation and also acts as an anchor for feedback. Exposing players to previous team action with videos may make performance monitoring expedient through precise feedback, which, in the next step, may make team member models more accurate.

The team leader’s failure to guide and structure team experiences to facilitate coordinative and adaptive action can be a key factor in ineffective team performance [50]. Deviant situational awareness between the majority of the players and the coach may be a major leadership threat, because it places greater demands on communication. Clarity in these expectations in specific game situations enables the team to adapt, and might increase leadership trust [25]. McComb [51] suggests that mental model convergence may be the key to understanding how individuals are transformed into team members, and the results show that the coach is on the same page as the majority of the players in all of the exposed videos, except video number two. To our knowledge, these facets of leadership in elite team ball games have not been considered in the literature and should be further elaborated on. This finding indicates that the coach and the players have similar preconditions to form accurate expectations for the task. Giving feedback and supervising players’ decisions in the game presuppose approximately identical situational awareness, otherwise coaching is about bringing similar situational awareness. By influencing situational awareness, the coach may also impact the players’ decision-making process [22]. This can be done, for example, by illuminating different options in the situations and clarifying priorities. To disclose players’ situational awareness, one should presume a dialogical coaching approach, where the players are invited to explore and verbalize their experiences from the game. Such a coaching practice seems to be in line with Salas et al. [25], who argue that team leadership affects team effectiveness, not by handing down solutions to the team, but by joint problem solving.

## 5. Conclusions and Practical Implications

The results from the present study reveal situations that point to the existence of shared situational awareness, whereas others display contradictory perceptions. However, being central or peripheral in the situation are conditions that also determine the possibility for adequate team coordination in soccer. According to Salas with colleagues [52], team training should only prioritize team competencies that yield the greatest impact on performance, and a major coaching task is therefore to uncover contradictory perceptions among the players in the most critical game situations. Exposing teams to videos from previous games, where the players individually express their opinion, may both improve their skills in monitoring team performance and strengthen their shared knowledge. The opinions from the players can uncover if the team has shared knowledge and in which situations there might exist discrepancies. Such information makes it possible to tailor teaching sequences directly towards situations where there are coordinative challenges. Furthermore, this approach gives information about each individual player’s cognition, and can therefore generate knowledge, which in the next step can be used to facilitate individual player development. Such a procedure is probably especially important with new players in the squad. However, further research is needed to confirm the usefulness of this approach. We encourage other researchers to investigate if our findings are comparable to other professional soccer teams. We would suggest a longitudinal approach to explore how teams develop and maintain shared situational awareness, and which methods in team training yield the best result. A main challenge in research with professional teams is getting access to do in-depth studies. Overcoming this obstacle would help expand our knowledge of cognitive factors in professional teams that are central in team coordination.

This study is not without its limitations, and these issues should be considered when the findings are interpreted. First, decision-making and situational awareness in sports is understood in a completely different way than in an ecological dynamics approach, where there is a direct link between perception and action [53]. This paper is, however, based on team literature, where knowledge or cognition is considered as essential, guiding players’ actions in some game situations. Second, the players and coach responded to twelve videos from one match. The selection of videos was based on the research group’s subjective perception of relevance. Other pictures from the match or other matches might have given a different result. However, the first author has been working as a professional with the responsibility of match analysis in a premier league club for several years, which should ensure both the relevance of the videos and trustworthiness in the interpretation of the data. In our opinion, the empirical material does “saturate” the phenomenon.

## Figures and Tables

**Table 1:** Participants’ experience, number of national and youth matches, interview length, and number of transcriptase pages.

ID	Elite Player Experience in Norway (Years) ^1^	National Matches	Youth National Matches	Number of Matches ^2^	Interview Length Time (min)	Transcript Pages
Player 1	13	No	Yes	120+	35	11
Player 2	3	No	Yes	40+	28	8
Player 3	7	No	No	140+	35	10
Player 4	3	Yes	Yes	70+	30	10
Player 5	5	No	Yes	100+	27	7
Player 6	15	Yes	Yes	300+	35	10
Player 7	6	Yes	Yes	70+	28	7
Player 8	4	No	Yes	20+	28	10
Player 9	2	No	No	30+	31	8
Player 10	3	No	No	70+	42	14
Coach					38	12

^1^ Years of experience in the premier league in Norway. ^2^ Exact information about the number of matches is not given due to the possibility of identifying the player.
